# Exploration of the Application of Data-Driven and Generation Models in the Design of Thermoplastic Vulcanizate Rubbers

**DOI:** 10.3390/polym17070995

**Published:** 2025-04-07

**Authors:** Hongyu Yang, Ce Hu, Yanhong Liu, Weimin Yang

**Affiliations:** 1 College of Mechanical and Electrical Engineering, Beijing University of Chemical Technology, Beijing 100029, China; 2020400151@buct.edu.cn; 2 Yantai Guogong Intelligent Technology Co., Ltd., Yantai 264006, China; ustchc317@gmail.com (C.H.); guogongsc@163.com (Y.L.); 3 State Key Laboratory of Organic-Inorganic Composites, Beijing University of Chemical Technology, Beijing 100029, China

**Keywords:** thermoplastic vulcanizate (TPV) rubber, data-driven, property prediction, generation model

## Abstract

The rapid advancement of big data and artificial intelligence has highlighted the substantial potential of data-driven approaches in polymer material research and development. In the present study, data-driven predictive models were developed to accurately forecast the density, tensile strength, flexural strength and melt mass flow rate of thermoplastic vulcanizate (TPV) rubber. Furthermore, a generation model was used to produce new material formula data for TPV rubber, and predictions were made for the aforementioned properties. The results indicated that the predicted values are in good agreement with experimental data. This study introduces innovative strategies and methodologies for the intelligent design of polymer materials, which could potentially lower research and development costs and accelerate the emergence of novel materials.

## 1. Introduction

Due to their excellent properties, polymer materials are widely used in many fields and play an indispensable and important role in the modern industrial system. Their light weight, high strength, corrosion resistance, easy processing and other characteristics allow them to play important roles in the aerospace, automobile, electrical, electronic device, medical and health care fields as well as daily necessities and other fields [1,2,3,4,5]. For a long time, traditional polymer materials research and development work mainly relied on the experience of experts and experimental trial and error, which not only consumed a lot of time and energy but also had low research and development efficiency and found it difficult to meet the needs for rapid development to satisfy increasing market demand [6,7,8]. 

In recent years, with the rapid improvement of computing power and the increasing maturity of high-throughput experimental technology, a large amount of theoretical and experimental data of polymer materials have been accumulated efficiently. These data cover a wealth of information in multiple dimensions from raw material selection and formulation ratios to processing parameters. Based on these massive data, the application of data-driven methods in polymer material science research has gradually received widespread attention. Data-driven methods help researchers better understand the complex relationship between material properties and preparation conditions by mining the intrinsic laws in the data. Meanwhile, machine learning and deep learning techniques have demonstrated significant advantages in dealing with nonlinear relationships and analyzing big data. These techniques can actively identify patterns and features in the data, providing strong support for the prediction and optimization of polymer material properties [9,10,11,12].

Thermoplastic vulcanizate (TPV) is a high-performance elastomer with a unique structural composition. It merges the outstanding resilience of conventional elastomer with the easy recyclability of thermoplastics. In TPV, crosslinked rubber exists as a dispersed phase of micron-sized rubber particles, while thermoplastic forms the continuous phase. Notably, the content of crosslinked rubber in TPV is higher than that of thermoplastics. In 1981, Coran of Monstanto Company in the United States prepared the first fully vulcanized ethylene propylene diene monomer (EPDM) rubber and polypropylene (PP) TPV by exploring experimental improvements in preparation methods [13]. The resulting product combines excellent physical and mechanical properties and processing properties. From then on, TPV began to be industrialized. To this day, EPDM/PP TPV is still the most common TPV product and is widely used in many fields such as the automotive, construction, and electronics industries [14,15,16,17,18].

Subsequently, researchers focused on the quantitative analysis of TPV. For instance, Thitihammawong et al. systematically studied the effects of different peroxide crosslinkers on the physical and mechanical properties of TPV [19]. The results showed that the type and amount of peroxide crosslinkers would affect the rheological, mechanical and morphological properties of TPV, which promoted the refined development of the TPV system. From a microscopic perspective, these properties are usually determined by the microstructure of TPV, which includes the rubber content, crosslinking degree, size of the rubber phase, uniformity of the rubber particle dispersion and structure of the rubber crosslinking network [20,21,22]. 

Kang et al. [23] used synthetic bio-based polyester elastomer (BPE) and polylactic acid (PLA) as the two components. They used an in situ dynamic crosslinking hybrid method to make a new bio-based TPV. As the rubber-phase content increased, the mechanical properties and hardness of TPV dropped a bit, yet its elasticity went up. The research by Wang’s group [24] shows that the difference between the rubber and plastic phases impacts the morphology and mechanical properties of TPV. When the weight ratio of ethylene vinyl acetate (EVA)/ethylene vinyl acetate rubber (EVM) TPV is 40/60, TPVs have great mechanical properties and no clear phase separation on the fracture surface. Cui et al. [25] reported that in hydrogenated acrylonitrile butadiene rubber (HNBR)/thermoplastic polyester elastomer (TPEE) TPVs, the melt flow rate of TPV becomes lower as the R/P ratio increases. The reason is that with more rubber, the rubber particles get bigger and form a stronger cross-linking network. This makes the processing performance of TPV worse. Therefore, the optimization of the formula that determines the microstructure is an important topic in the research and application of TPV materials [26,27]. A deep understanding of the correlation between TPV formulations and properties is important for the development of advanced materials that can be adapted to the needs of modern industry. This relationship not only affects the immediate performance of the material, but also its long-term stability and reliability under various operating conditions.

In this paper, based on the limited TPV actual formula data from the business partners of Yantai Guogong Intelligent Technology Co., Ltd., Yantai, China, the complex relationship between the formula and properties is explored through the development of a model, and new formulas are proposed based on this complex relationship model. Since the TPV produced here is mainly used in automotive parts, electronics, home appliances and daily necessities, this study only focused on four TPV properties that affect production and product quality, namely density, tensile strength, flexural strength and melt mass flow rate, which is an attempt to transform scientific research results into industry. Based on these production data, a machine learning model that can efficiently predict the four properties of TPV materials based on formula information was successfully constructed. This achievement not only provides a powerful tool for the optimization of properties of TPV materials, but also significantly improves the efficiency and accuracy of formula design. Further, the formula data set was expanded to two million for training formula generation models, so that it can generate more diverse formula combinations, providing a broader space for exploration for the research and development of new materials. On this basis, this paper combined the property prediction model and the generation model and used a reinforcement learning method to realize the design of new formulas. Reinforcement learning dynamically adjusts the formula generation model parameters and optimizes them according to the target properties to generate new formulas that are more in line with the needs. This method can not only quickly screen out formulas with excellent properties, but also reduce the cost of experimental trial and error. A series of research work in this paper verified the application potential of machine learning in the research and development of TPV materials. The described process from data-driven property prediction to new formula design based on reinforcement learning has also opened up a new path for the intelligent research and development of polymer materials.

## 2. Research Methodology

### 2.1. Data Acquisition and Preprocessing

The data used in this study are all derived from the formulation data of TPV polymer materials in actual industrial production, totaling 475 entries. These data cover formulation information, i.e., the proportions of raw materials, and the corresponding four property indicators: density, tensile strength, flexural strength, and melt mass flow rate. These property metrics are important parameters for assessing the processing performance of TPV materials and their performance in different application scenarios. In order to construct and validate the property prediction model as well as formulation generation model, the data were divided into three parts: 464 pieces of data for property prediction model training, 10 pieces of data for property prediction model testing, and 1 piece of data for validation of the formulation design of the generation model. This data division is intended to ensure that the model training, testing, and validation processes are independent of each other, thereby improving the generalization ability and reliability of the models.

**Figure 1:**
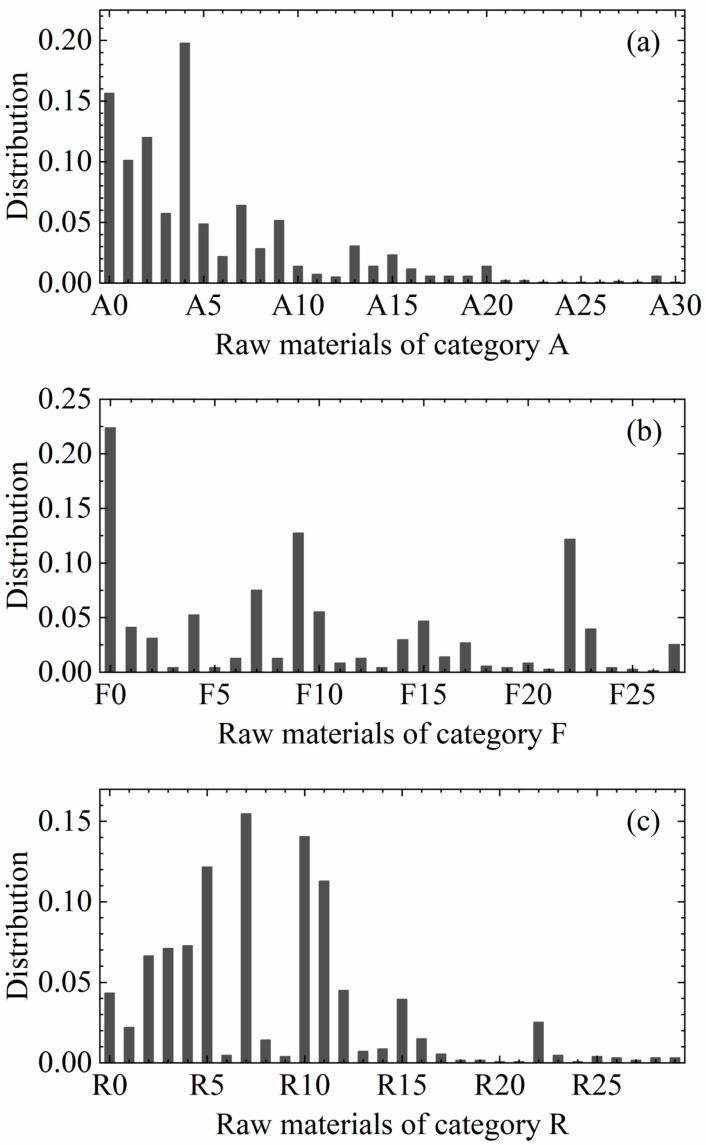
Frequency of occurrence of each ingredient in the formulation data. (**a**–**c**) indicate the distribution of ingredients in classes A, F and R, respectively. Ingredients in each class are labeled according to numbers, starting from 0.

TPVs are formulated with a wide range of raw materials, mainly including polyolefin materials to provide thermoplasticity, rubber materials to provide elasticity, crosslinking agents to initiate the vulcanization reaction of the rubber phase, as well as additives and modifiers, such as plasticizers, lubricants, flame retardants, pigments, etc., in order to adapt to the industrial production and a variety of application scenarios. For ease of presentation, the formulated ingredients in the data set were categorized into three classes in this paper, which are denoted by the labels A, F and R. Among them, A represents additives, such as plasticizers, fillers, antioxidants, lubricants, etc.; F represents functional agents, such as vulcanizing agents; and R represents basic raw materials, such as thermoplastic and rubber. There are 31 raw materials in category A, 28 raw materials in category F, and 30 raw materials in category R, totaling 89 raw materials. Figure 1 shows the distribution of the frequency of occurrence of each type of ingredient in all the formulation data. In all the formulation data, there are only 25 ingredients with more than 50 occurrences, while half of the ingredients have no more than 10 occurrences. This distribution indicates that the data in this training set are sparse and certain ingredients are used less frequently in the formulations, which may have some impact on the training and prediction properties of the model.

**Figure 2:**
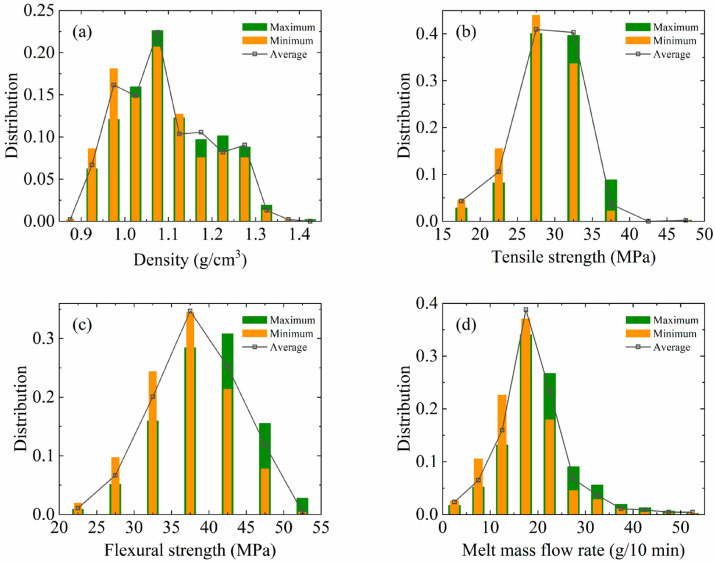
The distribution of four property values in the formulation data: (**a**) density, (**b**) tensile strength, (**c**) flexural strength and (**d**) melt mass flow rate.

Among the 475 formula data in this data set, 4014 property test data are included. Of these, 249 formulation data correspond to 1 test data. However, some formulations correspond to up to 20 property test data. These different property test data come from testing different batches of the same formulation. Figure 2 shows the distribution of the maximum, minimum and average values of the corresponding properties for each formulation. As can be seen from Figure 2, there is a significant difference in the distribution of properties tested for different batches of the same formulation. This reflects that the problem of accuracy of data collection in real industrial production is still not negligible. In order to improve the stability and reliability of the model, this paper adopted the average value of the property data of different batches as the property index of the corresponding formula. This method can reduce the impact of data fluctuation on model training to a certain extent and improve the adaptability of the model to different batches of data.

### 2.2. Property Prediction Model Construction

In order to accurately predict the four properties of TPV polymer materials, namely density, tensile strength, flexural strength and melt mass flow rate, the XGBoost algorithm [28] was used in this paper to construct the prediction model. XGBoost (extreme gradient boosting) is an efficient gradient boosting algorithm, which is widely used in various machine learning tasks. This algorithm progressively optimizes the prediction capabilities of the model by constructing multiple weak learners, usually decision trees. Each weak learner focuses on correcting the errors of the previous learner during the training process, thus gradually improving the prediction accuracy of the overall model. In this study, the parameters of the XGBoost model were carefully set to ensure that the model can effectively learn complex patterns in the data while avoiding overfitting. The specific parameters were set as follows: the learning rate was set to 0.05, which controls the contribution of each tree to the final model prediction results since a lower learning rate helps the model to learn the patterns in the data more robustly. The maximum depth of the tree (maxdepth) was set to 4, which restricts the complexity of each tree and helps to balance the model’s fitting ability and generalization ability. The number of estimators was set to 500 because a larger number of trees enables the model to capture the subtle changes in the data in a more detailed way, thus improving the prediction accuracy. The inputs to the model are sparse vectors based on ingredient ratios, with a vector length of 89, which corresponds to the proportions of the 89 different ingredients in the formula. Each element of the input vector represents the proportion of an ingredient in the formula, and these proportion values are normalized to ensure the stability and efficiency of model training. The outputs of the model are then property values corresponding to density, tensile strength, flexural strength and melt mass flow rate.

### 2.3. New Formula Generation Model

In order to realize the formula generation task, this paper adopted a stacked recurrent neural network (StackedRNN) [29,30] as the core architecture of the generation model. StackedRNN is formed by stacking multiple RNN layers together to form a complex sequence of data that can more effectively process the neural network architecture. The advantage of this architecture is its ability to capture long-term dependencies in sequence data, which is crucial for generating logical and rule-based formula strings. In the specific implementation process, this paper first processed the raw material ratio field in the dataset by converting it into a formula string field. To increase the diversity and complexity of the data, the order of the raw materials was randomly disrupted, thereby generating more formula combinations. In this way, the formula string data were expanded to 2 million, and the huge dataset provided rich material for the pre-training of the formula generation model, which enabled it to learn a variety of possible formula patterns and generate formula strings that conform to the formula laws.

**Figure 3:**
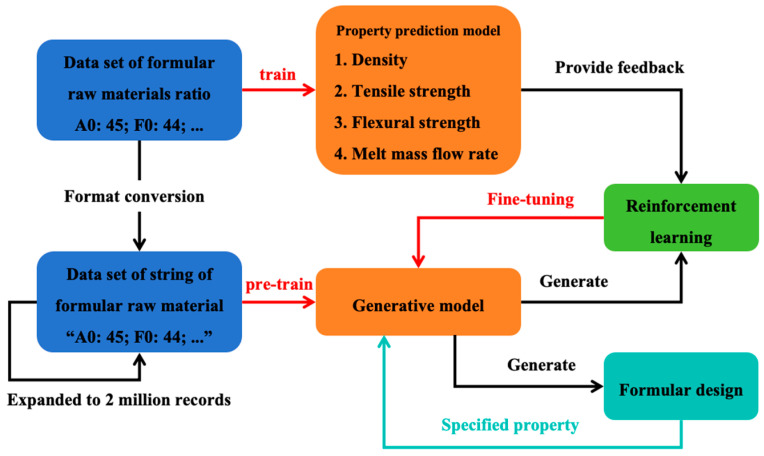
Schematic diagram of the formula generation process framework.

After pre-training was completed, the four property prediction models and the pre-trained formula generation model were integrated into the ACEGEN [31] reinforcement learning framework. The core objective of this framework was to fine-tune the parameters of the pre-trained models through the reinforcement learning algorithm while specifying the values of each target property. Specifically, the reinforcement learning algorithm guides the model to generate formulas that satisfy specific property requirements through a reward mechanism. In this process, the property prediction model provides feedback on property evaluation, while the generation model can adjust its own parameters based on such feedback to generate formulations that are closer to the target properties. This combination of property prediction and reinforcement learning framework not only generates rule-compliant formulas, but also ensures that the generated formulas can fulfill specific property requirements. The overall framework for formula generation is shown in Figure 3.

## 3. Results and Discussion

**Figure 4:**
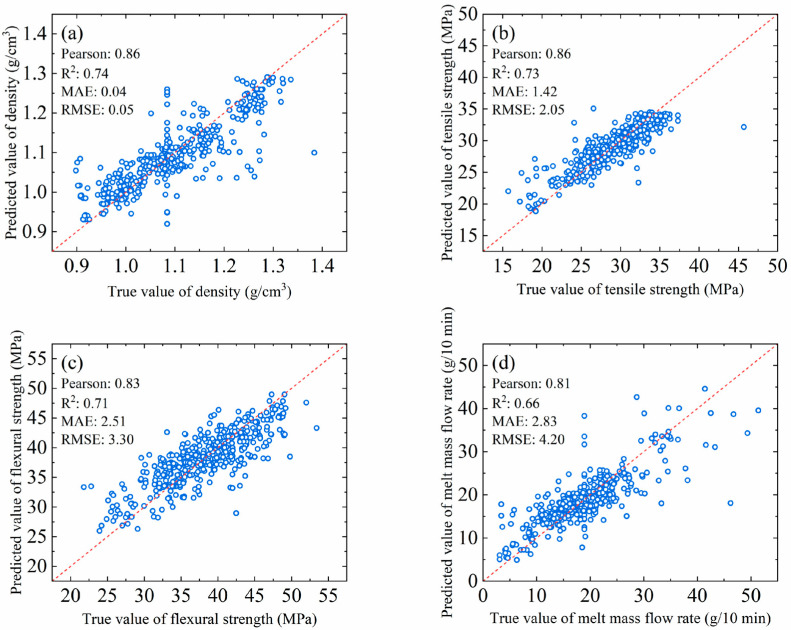
The training results of the four property prediction models: (**a**) density, (**b**) tensile strength, (**c**) flexural strength and (**d**) melt mass flow rate.

The results of the prediction model’s evaluation of the four properties of the training TPV material are shown in Figure 4: density, tensile strength, flexural strength and melt mass flow rate. It can be seen from Figure 4 that the Pearson correlation coefficients of the prediction models for density, tensile strength, bending strength and melt mass flow rate of the TPV material are 0.86, 0.86, 0.83 and 0.81, respectively; that is to say, the Pearson correlation coefficients of all the models are greater than 0.8, which indicates that there is a strong linear correlation between the predicted values of the model and the experimental values, and the model is able to capture the relationship between input features and target properties better. 

However, the Pearson correlation coefficient of the melt mass flow rate prediction model is slightly lower than those of the other three property models, which may be related to the complexity of the melt mass flow rate itself. The melt mass flow rate is not only affected by the material formulation but also closely related to the processing conditions and equipment parameters, and these complex factors make it necessary to consider more variables and interactions in the model prediction, which increases the difficulty of the model prediction. 

From the point of view of the coefficient of determination R^2^, the R^2^ values of all the property prediction models are greater than 0.7, except for the R^2^ of the melt mass flow rate prediction model (which is 0.66), which is slightly lower than 0.7. Specifically, the R^2^ values of the density, tensile strength and flexural strength prediction models are 0.74, 0.73 and 0.71, respectively, and these high R^2^ values indicate that the models are able to explain most of the experimental data and have a good fitting effect. Although the R^2^ value of the melt mass flow rate prediction model is slightly lower, it still has a certain predictive ability and can provide a reference for industrial production applications. 

Further, this paper evaluated the prediction error of the model from two perspectives: mean absolute error (MAE) and root mean squared error (RMSE). The results show that the MAE and RMSE of the density prediction model are smaller, 0.04 and 0.05, respectively. The MAE and RMSE of the tensile strength, flexural strength, and melt mass flow rate prediction models increase in this order but do not exceed a maximum of 3 and 4.5, respectively. These performances satisfy the needs of the industrial applications when compared to their respective ranges of values. Reasonable error values indicate that the differences between the predicted and experimental values are small and the model has a high prediction accuracy. These results further validate the effectiveness of the model in predicting TPV material properties.

**Figure 5:**
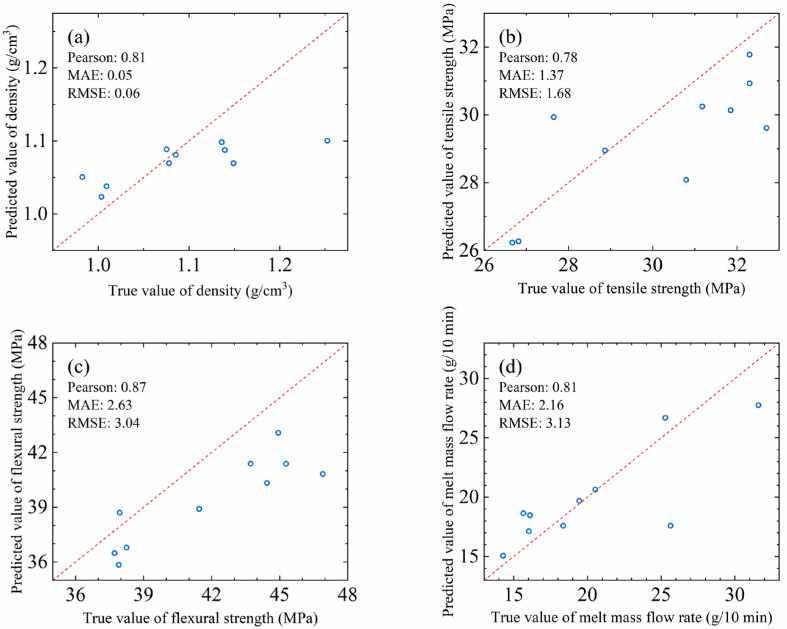
The performance of four property prediction models on the test data: (**a**) density, (**b**) tensile strength, (**c**) flexural strength and (**d**) melt mass flow rate.

In order to further verify the generalization ability and practical application value of the model, the obtained model was tested in this paper using an additional 10 formulation data as a test set, and the results are shown in Figure 5. The results showed that the Pearson correlation coefficients on the test set all exceeded 0.78, and the MAE and RMSE also differed from the predicted model to a lesser extent. Although the performance of the model on the test set was slightly lower than that on the training set, its prediction accuracy can still meet the requirements of industrial applications.

**Figure 6:**
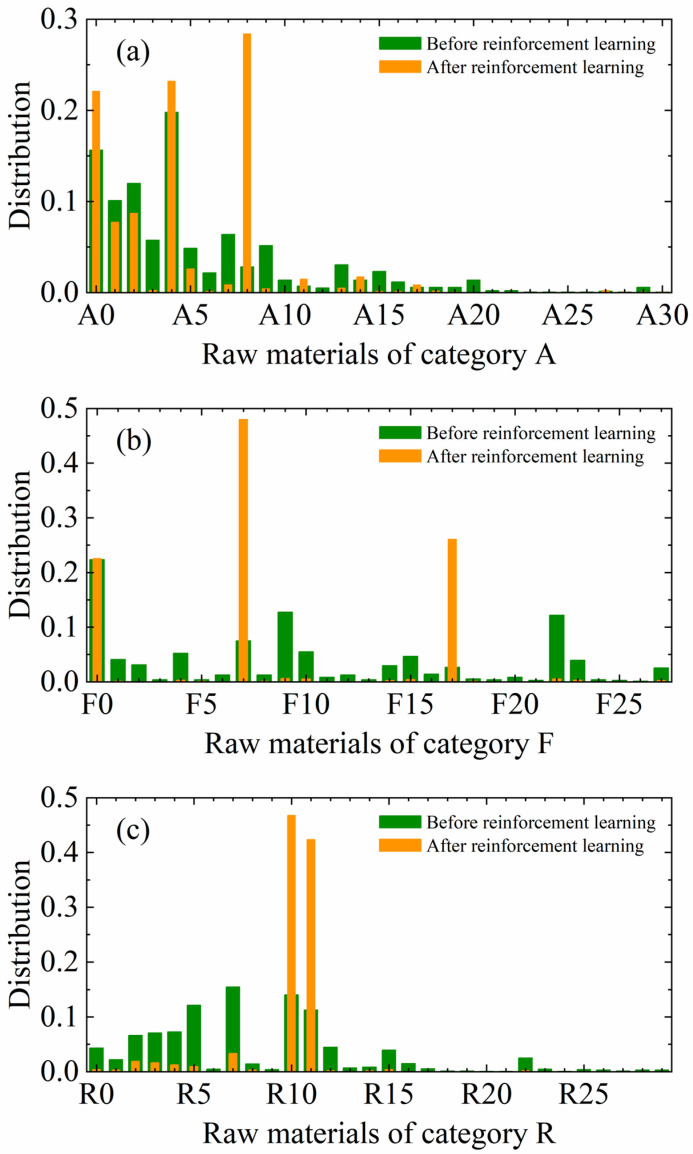
Difference in the frequency distribution of each type of ingredient in the generated formula before and after reinforcement learning. (**a**–**c**) indicate the distribution of ingredients in classes A, F and R, respectively. Ingredients in each class are labeled according to numbers, starting from 0.

After validating the effectiveness of the four property prediction models, this paper further explored the possibility of combining the generation model with the prediction model with a view to realizing the generation of formula data for a specified property target through reinforcement learning. The core of this process is to fine-tune the generation model using reinforcement learning algorithms so that it can generate the corresponding formula data according to the given property targets. Figure 6 clearly shows the changes in the distribution of the percentage of formula ingredients generated by the generation model before and after reinforcement learning. From the figure, it can be clearly seen that after the reinforcement learning training, the distribution of formula ingredients has changed significantly, which indicates that reinforcement learning can effectively guide the generation model to generate formulas that are close to the target properties.

In order to demonstrate the effect of this process more concretely, this paper selected one piece of formula data and set its corresponding four property values as target values. It should be emphasized that this recipe’s data did not participate in any previous model training. Based on these target values, two new formula datasets were generated in this paper using the generation model. These newly generated formula data not only satisfy the set target values in terms of properties, but also differ from the original formula in terms of raw material ratios. The specific results are shown in Table 1, which lists the original formula data and the two newly generated formula datasets, their corresponding property indexes, and the relative error between the predicted value and the real value. Through comparison, it can be found that the generated formulation 1 has adjusted the ratio of raw materials R11, F7 and A8, and additionally introduced new raw materials F15 and A0 while guaranteeing the target properties. And the generated formulation 2 has similar results. These results verify the effectiveness of reinforcement learning in formula generation, and the diversified raw material ratios also provide more choices and possibilities for the optimization of formulas in actual industrial production. The specific meaning of each symbol is shown in the notes of Table 1.

Theoretically, new formula data need to be verified in actual production to confirm the validity of each model in this paper. However, considering the difficulty of obtaining real experimental data, this is equivalent to doing a reverse verification here. It should be emphasized again that the recipe used for data generation did not participate in the training of any previous model, which is equivalent to an additional experiment. In the recipe generation process, only the four properties corresponding to the recipe were input into the generation model as target properties, without any recipe composition information. The effectiveness of the method was verified here mainly from two aspects: 1. The generated recipe was slightly different from the target recipe; 2. Under the premise of verifying the effectiveness of the four property prediction models in the above text, the property prediction values of the new recipe were slightly different from the actual property values of the target recipe.

## 4. Conclusions

In this paper, based on the data-driven method and with full consideration of the complexity of data in actual industrial production, efficient prediction models for four properties of TPV polymer materials, namely, density, tensile strength, flexural strength, and melt mass flow rate, were constructed and the StackedRNN model was used to generate new TPV material formulations based on reinforcement learning. The results showed that each prediction model constructed achieved good prediction accuracy on the test set, and the new formulations designed by the generation model are basically consistent with the experimental formulation data in terms of all four properties. This study demonstrated the feasibility of applying data-driven methods in the intelligent research and development of polymer materials, and provides an important reference for future multi-objective co-optimization, generation model improvement, and interdisciplinary applications.

In subsequent work, the error can be further reduced by tuning the hyperparameters of the model, increasing the number of training datasets, or adopting a more complex model structure. Moreover, when there is enough formula data, the data should be further finely divided according to the actual application scenarios of the formula, so as improve the correlation between the formula and the properties and the accuracy of the model. In addition, specific feature engineering and data preprocessing of the model by combining domain knowledge and expert experience will also help to improve the performance of the model. It should be noted that a high-quality and high-coverage data distribution is a prerequisite for building accurate models. Although the current data have covered most of the parameter space, there still exists the problem of data sparsity as well as insufficient data under certain extreme conditions. Therefore, in the future, high-throughput experimental techniques should be emphasized to further expand the data set or extend the existing data with data enhancement techniques to improve the robustness and generalization ability of the model.

## Figures and Tables

**Table 1:** Comparison of results between the generated and target formulas.

Actual formula	R10: 44.0; R11: 44.0; F7: 6.5; F0: 5.0; A8: 0.2; A1: 0.1; A2: 0.1				
Generated formula 1	R10: 44.0; R11: 40.0; F7: 8.0; F15: 7.5; A8: 0.3; A0: 0.2				
Generated formula 2	R10: 45.0; R11:40.0; F17: 5.5; F7: 5.0; F0: 4.0; A0: 0.2; A4: 0.2; A8: 0.1				
**Property**	**Real value**	**Predicted value 1**	**Predicted value 2**	**Error 1**	**Error 2**
Density	1.016	1.015	1.015	0.10%	0.10%
Tensile strength	35.6	34.6	34.5	2.81%	3.10%
Flexural strength	42.5	43.7	41.9	2.82%	1.41%
Melt mass flow rate	18.5	18.7	19.0	1.08%	2.70%

Notes: 1. Density, tensile strength, flexural strength and melt mass flow rate are given in g/cm^3^, MPa, MPa, and g/(10 min), respectively. 2. The specific meaning of the marks in the formula are: A0 for talcum powder, A1 for CaCO_3_, A2 for stearic acid, A4 for dioctyl phthalate (DOP), A8 for triallyl isocyanurate (TAIC), F0 for di-tert-butyl peroxide, F7 for dicumyl peroxide (DCP), F15 for benzoyl peroxide, F17 for phenolic resins, R10 for PP, and R11 for EPDM.

## Data Availability

Data are contained within the article.
